# Orthopædic Apparatus, by D. W. Kolbe

**Published:** 1869-02

**Authors:** 


ORTHOPÆDIC APPARATUS, and Description of Mechanical Apparatus employed in the Treatment of Deformities and Deficiencies of the Body, with Directions for taking Measurements for their Application. By D. W. KLOBE, Manufacturer of Surgical Instruments, and Machinist to the Philadelphia Orthopædic Hospital, No. 15 South Ninth street, Philadelphia.

This is the title of a neatly illustrated book, containing much useful information for all practitioners who have anything to do with orthopædic surgery. M. Klobe's skill as a machinist is undoubted, and as an instrument maker, he has few, if any, superiors. His catalogue will be sent to any physician who will furnish his address.

We have been in the habit of purchasing Surgical and Orthopædic Apparatus from M. Klobe for many years, and we take great pleasure in recommending him to all others wishing a good instrument and at reasonable prices. (See card.)

